# Stimulus-Specific Activation and Actin Dependency of Distinct, Spatially Separated ERK1/2 Fractions in A7r5 Smooth Muscle Cells

**DOI:** 10.1371/journal.pone.0030409

**Published:** 2012-02-21

**Authors:** Susanne Vetterkind, Robert J. Saphirstein, Kathleen G. Morgan

**Affiliations:** Department of Health Sciences, Boston University, Boston, Massachusetts, United States of America; Cardiff University, United Kingdom

## Abstract

A proliferative response of smooth muscle cells to activation of extracellular signal regulated kinases 1 and 2 (ERK1/2) has been linked to cardiovascular disease. In fully differentiated smooth muscle, however, ERK1/2 activation can also regulate contraction. Here, we use A7r5 smooth muscle cells, stimulated with 12-deoxyphorbol 13-isobutylate 20-acetate (DPBA) to induce cytoskeletal remodeling or fetal calf serum (FCS) to induce proliferation, to identify factors that determine the outcomes of ERK1/2 activation in smooth muscle. Knock down experiments, immunoprecipitation and proximity ligation assays show that the ERK1/2 scaffold caveolin-1 mediates ERK1/2 activation in response to DPBA, but not FCS, and that ERK1/2 is released from caveolin-1 upon DPBA, but not FCS, stimulation. Conversely, ERK1/2 associated with the actin cytoskeleton is significantly reduced after FCS, but not DPBA stimulation, as determined by Triton X fractionation. Furthermore, cytochalasin treatment inhibits DPBA, but not FCS-induced ERK1/2 phosphorylation, indicating that the actin cytoskeleton is not only a target but also is required for ERK1/2 activation. Our results show that (1) at least two ERK1/2 fractions are regulated separately by specific stimuli, and that (2) the association of ERK1/2 with the actin cytoskeleton regulates the outcome of ERK1/2 signaling.

## Introduction

Activation of extracellular signal regulated kinases 1 and 2 (ERK1/2) leads to a proliferative response in many cell types, including smooth muscle, where it has been connected with pathologic hypertrophy and hyperplasia, prerequisites for the development of hypertension and atherosclerosis [1], [2], [3], [4]. Furthermore, elevated ERK1/2 activity has been shown in atherosclerotic lesions of cholesterol-fed rabbits [5]. On the other hand, it has been demonstrated that ERK1/2 activation can regulate contractility in fully differentiated smooth muscle [6], [7], [8], and ERK1/2 has been shown to contribute to elevated tone in hypertensive rats [9].

ERK1/2 is activated by threonine and tyrosine phosphorylation by the dual specificity kinase MEK, which, in turn, is activated by Raf. Components of the MAPK cascade can be assembled by a variety of scaffold proteins, and these have been shown to regulate ERK1/2 cytoplasmic retention versus nuclear translocation (for review, see [10], [11]). However, subcellular targeting of ERK1/2 by scaffold proteins cannot fully explain the different outcomes of ERK1/2 activation, since even cytoskeletal ERK1/2 scaffolds have been associated with proliferative ERK1/2 signaling [12], [13]. Thus, it is still not clear how ERK1/2 activation can be directed towards proliferation in some settings, and towards contraction in others.

That ERK1/2 activity can be regulated by caveolin-1 is widely agreed upon, however, both activation and inhibition of ERK1/2 by caveolin-1 have been reported [14], [15], [16], [17], [18], [19]. Caveolin-1 is the characteristic membrane protein in flask-like membrane invaginations, caveolae. It is expressed at high levels in contractile smooth muscle, and high expression levels correlate with and, moreover, support the contractile phenotypic state of smooth muscle cells [20]. Therefore, caveolin-1 is a good candidate for mediating contractile, as opposed to proliferative, ERK1/2 stimulation in smooth muscle.

In the present study we use, as a model system, the smooth muscle derived A7r5 cell line to investigate what factors determine the outcome of ERK1/2 signaling in smooth muscle cells. A7r5 cells re-acquire a smooth muscle-like phenotype, as determined by the upregulation of smooth muscle marker proteins such as alpha actin, basic calponin and SM22 after growth to confluency and serum starvation [21], [22], [23]. ERK1/2 activation was induced either by the phorbol ester, 12-deoxyphorbol 13-isobutylate 20-acetate (DPBA), or by fetal calf serum (FCS) stimulation. These two stimuli, although both induce ERK1/2 activation, produce very different effects: DPBA triggers ERK1/2 dependent cytoskeletal remodeling and formation of podosomes [24], [25], whereas FCS induces proliferation [26]. Using this model system we report here that different pools of ERK1/2 exist in the same cell type and that they are differentially affected by specific stimuli. Phorbol esters release activated ERK1/2 from caveolae in an actin dependent manner, and simultaneously activate cytoskeletal ERK1/2, whereas a proliferative stimulus does not affect caveolar ERK1/2 and releases ERK1/2 from actin to trigger proliferative outcomes.

## Results

### Caldesmon phosphorylation in response to a phorbol ester depends on ERK1/2

**Figure 1:**
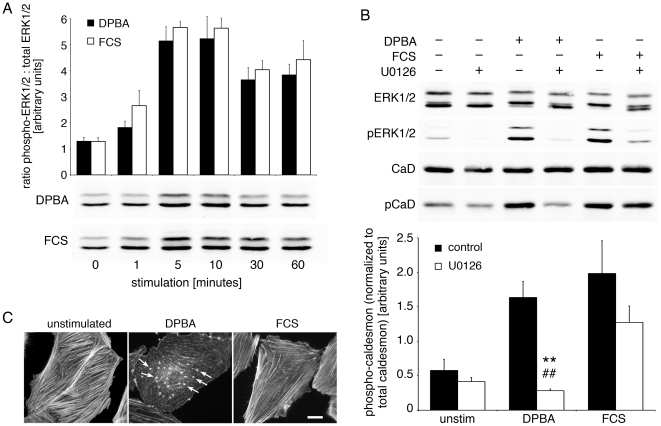
ERK1/2 activation has stimulus-specific effects on the A7r5 cytoskeleton. (A) ERK1/2 is phosphorylated in response to both, DPBA and FCS. A7r5 cells stimulated with either DPBA or FCS for the indicated time points were processed for western blot analysis of total ERK1/2 and phospo-ERK1/2. After densitometry analysis, no significant differences in ERK1/2 phosphorylation (normalized to total ERK1/2) were detected. (B) A7r5 cells were pre-treated with the MEK inhibitor U0126 for 60 minutes, then stimulated with DPBA or FCS, or left untreated, for additional 60 minutes before preparation of cell lysates. Lysates were analyzed by western blotting and densitometry. Please note that in the presence of the MEK inhibitor, caldesmon phosphorylation in response to DPBA is completely blocked, whereas caldesmon phosphorylation (normalized to tubulin) in response to FCS is reduced by only about 30%. Statistical significance was tested by a two-tailed Student's T-Test (**p<0.01 U0126 vs. control, ^##^p<0.01 DPBA vs. FCS). (C) A7r5 cells grown on coverslips were treated as indicated for 60 minutes, then fixed and stained for fluorescence microscopy with phalloidin to visualize actin filaments. Please note the cytoskeletal rearrangements and the appearance of podosomes in the DPBA treated cells (arrows). Scale bar, 20 µm.

ERK1/2 is phosphorylated after both DPBA and FCS stimulation of A7r5 smooth muscle cells (Figure 1A). Phosphorylation of the actin-binding ERK1/2 substrate caldesmon has been shown to mediate podosome formation in response to phorbol ester stimulation of protein kinase C through activation of ERK1/2 [25]. Therefore, to test whether caldesmon mediates the different effects of DPBA versus FCS stimulation, we analyzed caldesmon phosphorylation in response to these two stimuli. Interestingly, our results show that caldesmon, like ERK1/2, is phosphorylated in response to both DPBA and FCS stimulation. However, DPBA induced phosphorylation of caldesmon fully depends on ERK1/2 activation, since in the presence of the MEK inhibitor U0126, caldesmon phosphorylation is reduced to background levels. In contrast, after FCS stimulation, caldesmon phosphorylation is reduced by roughly 30% in the presence of U0126, indicating that caldesmon phosphorylation is only partly mediated by ERK1/2 after FCS stimulation (Figure 1B). Yet, although in both types of stimulation ERK1/2 and caldesmon are phosphorylated, DPBA stimulation leads to podosome formation, while FCS stimulation does not (Figure 1C). These observations prompted us to test whether specific ERK1/2 subfractions are activated in a stimulus specific manner.

### Caveolin-1 mediates ERK1/2 activation in response to phorbol ester, but not serum stimulation

**Figure 2:**
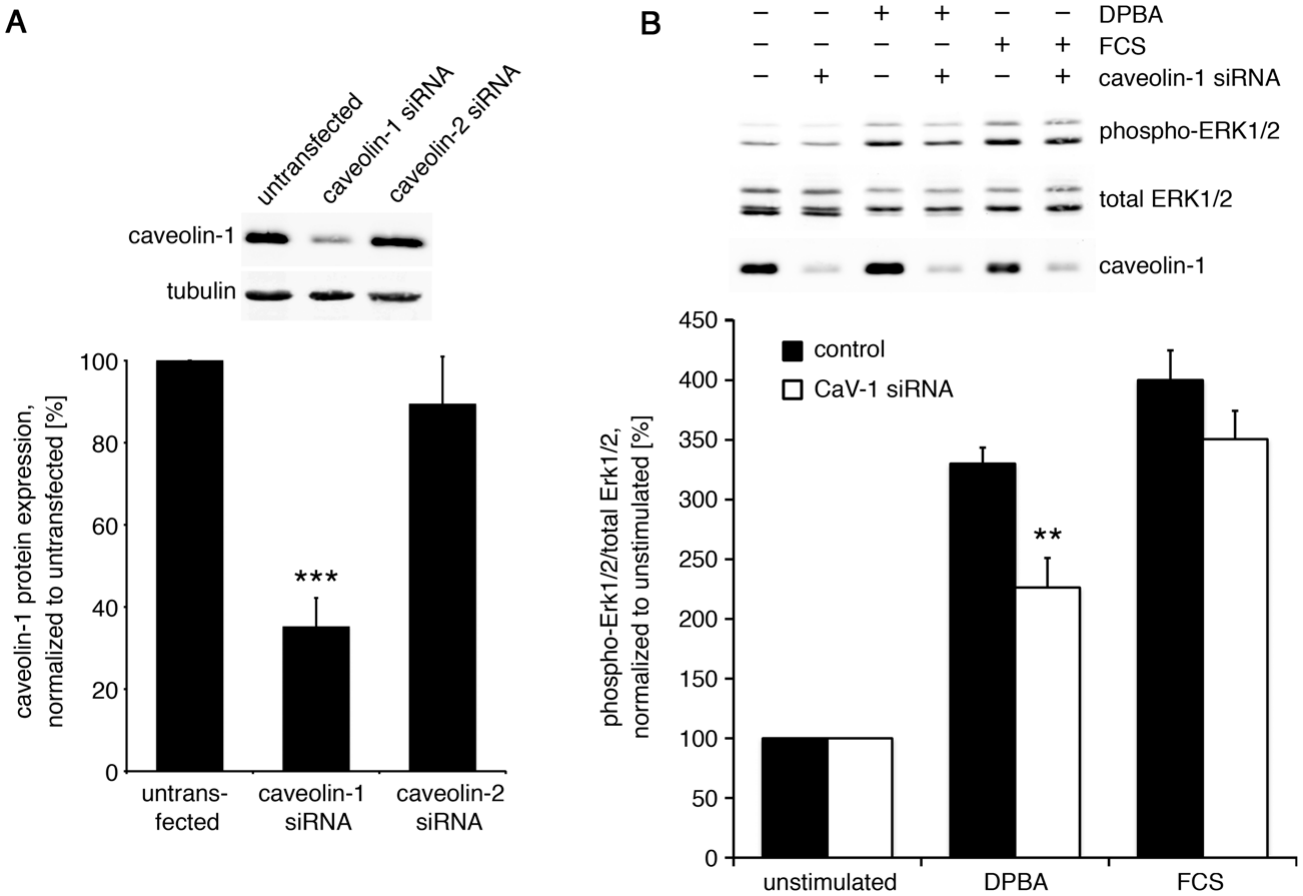
Knock down of caveolin-1 interferes with DPBA induced ERK1/2 phosphorylation. A7r5 cells were transfected with caveolin-1 siRNA, caveolin-2 siRNA as control or left untransfected. Experiments were performed five days after transfection. (A) Western blot analysis and statistical analysis of densitometry analysis show efficient knock down of caveolin-1. Statistical significance was tested by two-tailed Student's T-Tests (***p<0.001 siRNA vs. control, ^###^p<0.001 siRNA vs. untransfected). (B) Five days after siRNA transfection, cells were stimulated with either DPBA or FCS, or left untreated. Lysates were analyzed for ERK1/2 phosphorylation by western blotting and densitometry. The graph shows that after caveolin-1 knock down, the rate of ERK1/2 phosphorylation is significantly reduced in response to DPBA, but not FCS. Statistical significance was tested by two-tailed Student's T-Tests (**p<0.01 siRNA vs. control, ^##^p<0.01 DPBA vs. FCS).

Caveolin-1 has been shown to act as an ERK1/2 scaffold in smooth muscle, and interference with caveolin-1 signaling in a decoy peptide approach reduced the contraction of smooth muscle tissue after PKC-mediated contractile stimulation [16]. With the help of siRNA experiments to knock down endogenous caveolin-1 expression, we have tested the hypothesis that caveolar ERK1/2 is affected differently by stimuli that lead to cytoskeletal remodeling (DPBA) or proliferation (FCS). Control experiments demonstrate that caveolin-1 siRNA reduces the expression of caveolin-1 by approximately 65%, whereas control siRNA against caveolin-2 (which is not expressed at detectable levels in A7r5 cells) does not affect caveolin-1 expression (Figure 2A). Western blot followed by densitometry analysis revealed that after siRNA knock down of caveolin-1, ERK1/2 phosphorylation (normalized to total ERK1/2) in response to DPBA, but not FCS, is significantly reduced by approximately 30% (Figure 2B). In the caveolin-1 siRNA treated cells, but not the control cells, ERK1/2 phosphorylation in response to DPBA is also significantly reduced when DPBA and FCS stimulated groups were compared. These findings indicate that caveolin-1 promotes ERK1/2 activation in a stimulus-specific manner. Separate analysis of ERK1 and ERK2 shows that the effect of caveolin-1 siRNA was more pronounced for ERK2 than for ERK1. Significance was reached for the DPBA stimulated CaV-1 siRNA samples compared to FCS stimulated CaV-1 siRNA samples (p<0.014) as well as compared to DPBA stimulated control samples (p<0.015). ERK1 is also reduced in the CaV-1 knock down samples, but the difference is not quite statistically significant.

### A caveolar ERK1/2 fraction is phosphorylated and released after phorbol ester stimulation

**Figure 3:**
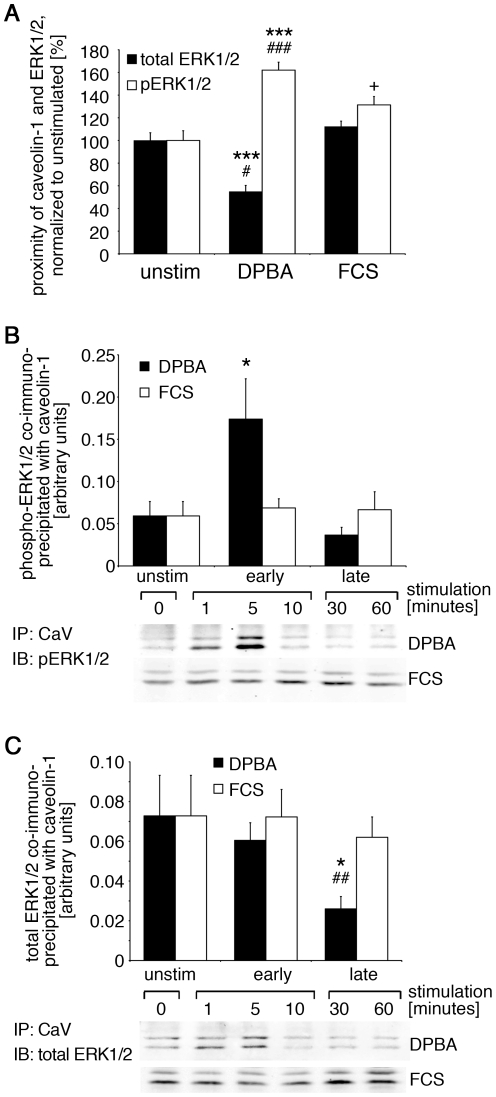
A caveolar fraction of ERK1/2 is phosphorylated and mobilized in a stimulus-specific manner. (A) A7r5 cells grown on coverslips were stimulated with DPBA or FCS, or left unstimulated, and then fixed and stained with a caveolin-1 antibody and either an ERK1/2 or phospho-ERK1/2 antibody. Cells were processed for proximity ligation assays (Olink) according to the manufacturer's protocol and then analyzed for signal dots indicative of close proximity. (B and C) A7r5 cells were treated with either DPBA or FCS for the indicated times. Lysates were then subjected to immunoprecipitation with an anti-caveolin-1 antibody. Immunoprecipitates were analyzed for co-immunoprecipitation of (B) phospho-ERK1/2 and (C) total ERK1/2 by western blotting and densitometry. Please note that interaction of caveolin-1 with phospho-ERK1/2 is increased at early time points after DPBA, but not FCS stimulation, whereas interaction with total ERK1/2 is reduced at later time points of DPBA, but not FCS stimulation. Statistical significance was tested by two-tailed Student's T-Tests (*p<0.05, ***p<0.001 DPBA vs. unstimulated; ^#^p<0.05, ^##^p<0.01, ^###^p<0.001 DPBA vs. FCS; ^+^p<0.05 FCS vs. unstimulated).

To investigate how the interaction of caveolin-1 and ERK1/2 is affected by DPBA versus FCS stimulation, we first utilized proximity ligation assays (Olink, Uppsala, Sweden) with anti-caveolin-1 and anti-ERK1/2 specific antibodies (Figure 3A). Data obtained from these experiments show that proximity of caveolin-1 with phospho-ERK1/2 is markedly enhanced after 10 minutes of DPBA stimulation and slightly enhanced after 10 minutes FCS stimulation. At the same time point, proximity of caveolin-1 and total ERK1/2 is significantly reduced after DPBA stimulation, but not affected by FCS stimulation. These results suggest that a caveolar fraction of ERK1/2 is released from caveolae upon ERK1/2 phosphorylation after DPBA, but not after FCS stimulation. To investigate the time course of ERK1/2 and caveolin-1 interaction in more detail, we performed IP experiments with an anti-caveolin-1 antibody. Time points analyzed were 0 minutes, 1 minute, 5 minutes, 10 minutes, 30 minutes and 60 minutes, but for clarity, data are grouped into unstimulated (0 minutes), early (1–10 minutes) and late (30–60 minutes), with the early timepoints comprising the observed peak of ERK1/2 phosphorylation (Figure 1A) between 5 and 10 minutes. Co-immunoprecipitates were analyzed for phospho-ERK1/2 (Figure 3B) and total ERK1/2 (Figure 3C), both normalized to immunoprecipitated caveolin-1. Phospho-ERK1/2 was found to co-precipitate at early time points after DPBA stimulation with the strongest co-precipitation detected between 5 and 10 minutes, but not at later time points. For total ERK1/2, a small non-significant increase of binding to caveolin-1 was observed early after DPBA stimulation, however, at later time points the association of ERK1/2 and caveolin-1 was significantly reduced, compared to both, unstimulated and FCS stimulated. In contrast, no significant change in binding of phosphorylated or total ERK1/2 was found after FCS stimulation. The results confirm that the fraction of ERK1/2 that is associated with caveolin-1 under unstimulated conditions can be phosphorylated and subsequently released in a stimulus-specific manner.

### Filamentous localization of phospho-ERK1/2 is lost after caveolin-1 knock down

**Figure 4:**
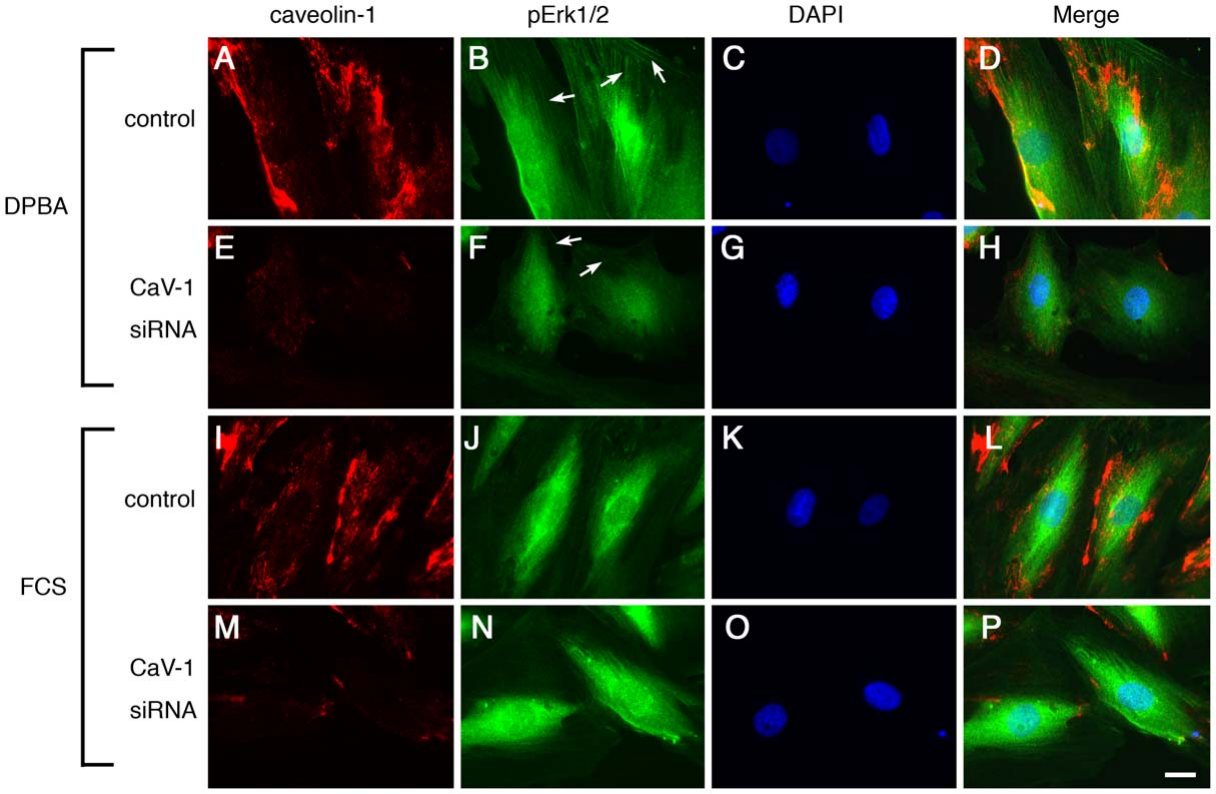
Stimulus-specific cytoskeletal localization of phospho-ERK1/2 depends on caveolin-1. A7r5 cells grown on coverslips were transfected with caveolin-1 siRNA and control siRNA. Five days after transfection, cells were stimulated with either DPBA or FCS for 60 minutes, or left untreated. After stimulation, cells were fixed and stained for caveolin-1 (a, e, I, m), phospho-ERK1/2 (b, f, j, n) and DAPI (c, g, k, o); merged images are shown in panels d, h, l, p. Please note that in the caveolin-1 knock down cells, the filamentous phospho-ERK1/2 staining seen after DPBA stimulation is absent in caveolin-1 knock down cells (arrows). Scale bar, 20 µm.

The data from the knock down and IP experiments suggest that the reduction in ERK1/2 phosphorylation after caveolin-1 knock down represents the caveolar ERK1/2 fraction only. We therefore applied immunofluorescence analysis of phospho-ERK1/2 to show whether the effect of caveolin-1 knock down on ERK1/2 phosphorylation is localization dependent (Figure 4). Surprisingly, imaging with a phospho-specific ERK1/2 antibody revealed that phospho-ERK1/2 shows stimulus-specific staining patterns, with DPBA causing a filamentous staining pattern (B) and FCS resulting in diffuse, mainly perinuclear staining (J). Furthermore, after caveolin-1 siRNA treatment, the ERK1/2 phosphorylation in response to DPBA was reduced and filamentous staining was lost (F). FCS-induced ERK1/2 phosphorylation was not affected (N), as expected from the results presented in Figure 1B. Co-staining of DPBA treated cells with phospho-ERK1/2 and either alpha smooth muscle actin or phalloidin to visualize actin filaments confirmed that filamentous ERK1/2 staining colocalizes with actin stress fibers (Supporting Information S1).

### Insoluble ERK1/2 fractions are activated and mobilized in a stimulus-specific manner

**Figure 5:**
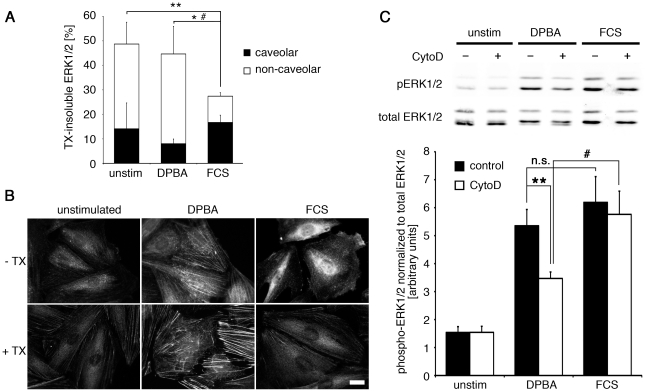
The cytoskeletal fraction of ERK1/2 is mobilized upon FCS, but not DPBA, stimulation. (A) A7r5 cells were treated with either DPBA or FCS and then subjected to subcellular fractionation into cytoskeletal and soluble fractions by Triton X-100 extraction. ERK1/2 subcellular distribution was analyzed by western blotting and densitometry. The graph shows the percentage of total ERK1/2 found in the caveolar and non-caveolar (cytoskeletal) TX-insoluble fractions at late time points of stimulation (30 minutes and 60 minutes). Statistical significance was tested by two-tailed Student's T-Tests (non-caveolar insoluble fraction: *p<0.05 DPBA vs. FCS, **p<0.01 unstimulated vs. FCS; caveolar insoluble fraction: ^#^p<0.05 DPBA vs. FCS). (B) A7r5 cells were grown on coverslips were subjected to TX extraction before fixing and staining for phospho-ERK1/2. Please note that filamentous phospho-ERK1/2 staining after DPBA stimulation is TX resistant, while the nuclear and diffuse perinuclear phospho-ERK1/2 staining after FCS stimulation is not. Scale bar, 20 µm. (C) A7r5 cells, pretreated with Cytochalasin D for one hour or control treated (solvent only), were stimulated with either FCS or DPBA, or left unstimulated, for another hour. Lysates were subjected to western blotting with a total ERK1/2 antibody and a phospho-ERK1/2 antibody. The graph shows phospho-ERK1/2 relative to total ERK1/2. Statistical significance was tested by a paired two-tailed Student's T-Test (**p<0.01 Cyto D vs. control, #p<0.05 DPBA vs. FCS, n.s. = not significant).

Since in the immunofluorescence experiments, a caveolar fraction of ERK1/2 is not clearly identifiable due to resolution limitations, we used a Triton X-100 (TX) extraction/western blot approach to analyze what fraction of ERK1/2 is associated with caveolae and how it is affected by stimulation. The caveolar fraction is insoluble at 4°C, but not at 37°C, whereas the cytoskeletal fraction is insoluble at both conditions. Experiments were carried out in parallel at 4°C and at 37°C to allow for calculation of the caveolar fraction and the non-caveolar, presumably mainly cytoskeletal fraction. Surprisingly, as shown in Figure 5A, almost 50% of total ERK1/2 was found in the insoluble fraction of unstimulated cells, most of it non-caveolar. In response to serum stimulation, ERK1/2 showed increased solubility and was significantly reduced in the insoluble fraction, while the amount of ERK1/2 in the caveolar fraction was unchanged, indicating that ERK1/2 is recruited from the cytoskeleton, but not from caveolae, for nuclear translocation after serum stimulation. DPBA stimulation reduced the amount of caveolar ERK1/2 by 50%, as expected from the experiments shown in Figure 3, but had little effect on the non-caveolar insoluble ERK1/2 fraction, suggesting that the cytoskeletal fraction of ERK1/2 remains bound to the cytoskeleton and is not released in response to DPBA. The results shown in Figure 5A represent data from late time points of stimulation (30 and 60 minutes), however, analysis of early time points (1–10 minutes) also revealed a significant reduction of the non-caveolar insoluble fraction after FCS stimulation (compared to unstimulated and DPBA stimulated), and a significant smaller caveolar fraction after DPBA stimulation compared to FCS stimulation. These results are corroborated by immunofluorescence imaging of phospho-ERK1/2 in DPBA or FCS stimulated (60 minutes) or unstimulated cells with and without TX-extraction at 37°C before fixing. As shown in Figure 5B, the filamentous phospho-ERK1/2 staining observed after DPBA stimulation is resistant to TX extraction. In contrast, the nuclear and diffuse perinuclear phospho-ERK1/2 staining that is seen after FCS stimulation is lost after TX-extraction.

### The actin cytoskeleton is required for ERK1/2 activation in response to a phorbol ester

To further evaluate the role of the actin cytoskeleton in channeling of ERK1/2 signaling, we analyzed ERK1/2 phosphorylation after disruption of the actin cytoskeleton (Figure 5C). To this end, cells were pretreated with the inhibitor of actin polymerization, cytochalasin D before stimulation with serum or DPBA for 60 minutes. The results show that cytochalasin D reduces ERK1/2 phosphorylation in response to DPBA by approximately 40%, but has no significant effect on serum-mediated ERK1/2 phosphorylation, demonstrating that an intact actin cytoskeleton is essential for activation of ERK1/2 after DPBA stimulation.

## Discussion

**Figure 6:**
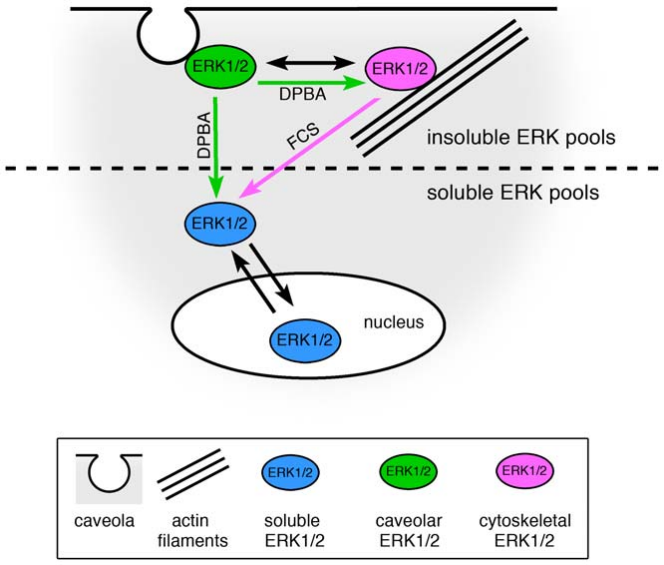
Model: Spatially distinct ERK1/2 subfractions are activated in a stimulus-specific manner. Upon FCS stimulation, a cytoskeletal ERK1/2 fraction is activated and released into a soluble ERK1/2 fraction (magenta arrow), enabling nuclear ERK1/2 signaling, whereas the caveolar ERK1/2 fraction is not activated in response to FCS. Phorbol ester stimulation, in contrast, leads to phosphorylation of both, caveolar and cytoskeletal ERK1/2 fractions. Caveolar ERK1/2 is released upon activation (green arrows), while cytoskeletal ERK1/2 is retained at actin filaments. The double headed arrow indicates a functional link between the caveolar and cytoskeletal ERK1/2 fractions.

The main finding of the present study is that in smooth muscle cells, physically distinct ERK1/2 subfractions are stimulated separately by specific stimuli. Based on our data, we suggest that at least two insoluble ERK1/2 fractions exist (see model in Figure 6), namely a caveolar and a cytoskeletal ERK1/2 fraction, and that phorbol ester stimulation mobilizes the caveolar pool and activates the cytoskeletal pool, whereas serum stimulation leads to a release of cytoskeletal ERK1/2 into the soluble ERK1/2 pool.

The precise role of caveolin-1 and caveolae as scaffolds for MAPK signaling is still under debate. Many groups have reported enhanced ERK1/2 activation after cholesterol depletion to remove caveolae, or after knock down of caveolin-1, indicating an inhibitory role for caveolae in MAPK signaling [14], [15], [17]. In smooth muscle, caveolin-1 can direct growth factor signaling to either a proliferative or an apoptotic response [18]. Furthermore, caveolin-1 has been shown to regulate smooth muscle contractility [16], [19]. Our findings suggest that in smooth muscle, caveolae can both, sequester ERK1/2 molecules to withdraw them from the soluble pool, and facilitate ERK1/2 signaling, depending on the stimulus. This points to a trendsetting function of caveolae in inhibiting proliferative ERK1/2 signaling, while at the same time facilitating cytoskeletal ERK1/2 signaling, which is in line with the positive correlation between differentiation status and expression level of caveolin-1 in smooth muscle [20] and also explains the apparently contradictory roles that caveolae seem to play in MAPK signaling.

The caveolar ERK1/2 fraction, that was activated and mobilized in response to DPBA, represented a relatively small percentage of total ERK1/2 of about 10%, which was surprising in light of the well known function of caveolin-1 as an ERK1/2 regulator. This number is feasible, though, given that catalytic amounts of ERK are likely sufficient to have significant downstream effects because of signal amplification. However, it might also be an underestimation of the real ERK1/2 percentage in the caveolar fraction, since in density gradient fractionations, roughly 20% of total ERK1/2 was found in the caveolar fraction (Supporting Information S1), indicating that not all of the caveolar ERK1/2 is pulled down together with caveolin-1 in IP experiments. Due to the small size of the caveolar ERK1/2 fraction, we were not able to resolve where activated caveolar ERK1/2 is targeted after mobilization. However, the observed dependence of activation of cytoskeletal ERK1/2 on caveolin-1 (Figure 4) and thus, on functional caveolae, suggests a link between the caveolar and cytoskeletal ERK1/2 fractions. Therefore, it seems likely that activated caveolar ERK1/2 is targeted to the cytoskeleton.

An unexpectedly large percentage of total ERK1/2 was found in a Triton X insoluble cytoskeletal compartment in unstimulated cells, and after DPBA stimulation, phospho-ERK staining displayed a filamentous pattern indicative of cytoskeletal association. Although ERK1/2 has several cytoskeletal interaction partners and substrates [27], [28], [29], [30], there are few reports about cytoskeletal association of ERK1/2, most of them showing ERK1/2 in a Triton X-insoluble fraction in protein biochemistry experiments [31], [32], [33], [34]. Of note, ERK1/2 has been shown to bind to actin directly in vitro [32]. Imaging of phospho-ERK1/2 associated with the actin cytoskeleton has, to the best of our knowledge, only been shown for NIH3T3 cells [35] and vascular smooth muscle cells [36], [37], but the functional significance of actin association of ERK1/2 has, so far, not been investigated in detail. We show here that in smooth muscle cells (1) a large fraction of total ERK1/2 is associated with the actin cytoskeleton in a Triton X-insoluble manner, (2) is targeted differently by different stimuli, (3) can be activated in a caveolin-1 dependent manner, and (4) provides an important actin-dependent fraction of activated ERK1/2 after phorbol ester stimulation, thus clearly linking ERK1/2 function to the actin cytoskeleton.

Immobilization of ERK1/2 in a cytoskeletal pool could account for some of the differences in ERK1/2 signaling that have been observed in smooth muscle cells [6], [7], [8] versus other cell and tissue types [38], [39], [40], and also for the different directions of ERK1/2 signaling (contraction versus proliferation) in fully differentiated, contractile smooth muscle versus synthetic, non-contractile smooth muscle [41], [42]. It is tempting to speculate that, since contractile smooth muscle contains high levels of actin and other cytoskeletal proteins, the protein equipment of contractile cells could directly channel ERK1/2 activation towards cytoskeletal targets and thus, diminish nuclear ERK1/2 signaling. However, in the experiments presented here, ERK1/2 subcellular localization does not reflect dynamic changes of the actin cytoskeleton: although DPBA stimulation leads to reorganization of actin filaments in favor of podosome formation, the cytoskeletal ERK1/2 fraction remains associated with the actin cytoskeleton. Conversely, after FCS stimulation, stress fibers remain intact (Figure 1A), yet ERK1/2 dissociates from the cytoskeletal fraction upon FCS stimulation. These findings suggest that cytoskeletal association of ERK1/2 is most likely regulated by other, stimulation-dependent factors, rather than protein levels of actin or actin binding proteins.

In summary, we show here for the first time that in smooth muscle, the outcome of ERK1/2 activation depends on the type of stimulation and is determined by cytoskeletal binding of ERK1/2: in response to serum, which mimics exposure to serum growth factors after blood vessel injury, initially insoluble ERK1/2 is mobilized, enabling nuclear signaling. In contrast, signaling downstream of phorbol ester stimulation, which mimics vasoconstrictor-induced activation of protein kinase C [43], depends on insoluble caveolar and cytoskeletal ERK1/2 pools, resulting in cytoskeletal remodeling. Thus, our results identify cytoskeletal association of ERK1/2 a mechanism by which ERK1/2 signaling can be directed either toward proliferation, as associated with atherosclerosis [1], or toward contractility, as associated with hypertension [9].

## Materials and Methods

### Reagents and antibodies

General laboratory reagents were of analytical grade or better and were purchased from Sigma (St. Louis, MO) and Bio-Rad (Hercules, CA). For stimulation, fetal calf serum (FCS, Invitrogen, Carlsbad, CA) was used at 10% and 12-deoxyphorbol 13-isobutylate 20-acetate (DPBA, LC Laboratories, Woburn, MA) was used at 3 µM. The MEK inhibitor U0126 (Calbiochem, Gibbstown, NJ) was used at 20 µM. Cytochalasin D (CytoD, Cytoskeleton, Denver, CO) was used at 5 µM, as determined in test experiments as optimal concentration for effective disruption of the cytoskeleton. The following primary antibodies were used: rabbit polyclonal anti-caveolin-1 (Cell Signaling, Danvers, MA), mouse monoclonal anti-ERK1/2 (Cell Signaling), mouse monoclonal anti-phospho-ERK1/2 (Cell Signaling), rabbit polyclonal anti-phospho-ERK1/2 (Cell Signaling), mouse monoclonal anti-tubulin alpha (Sigma), rabbit polyclonal anti-phospho-caldesmon (Millipore, Billerica, MA), rabbit polyclonal total caldesmon (Abgent, San Diego, CA). For immunofluorescence experiments, goat anti-rabbit and goat anti-mouse Alexa Fluor® 488 and Alexa Fluor® 568 (1∶1000, Invitrogen) were used as secondary antibodies. Goat Oregon Green® 488 or Alexa Fluor® 568 labeled anti-rabbit or anti-mouse IgGs were used as secondary antibodies in western blot experiments (1∶1000, LI-COR, Lincoln, NE). Nuclei were stained with 4,6-diamidino-2-phenylindole (DAPI, Sigma), and filamentous actin was stained with Alexa Fluor 568 phalloidin (1∶3000, Invitrogen).

### Cell culture

A7r5 rat aorta cells (ATCC, Manassas, VA) were cultured in DMEM high glucose (Invitrogen) with 10% FCS, 1% glutamine, 50 units/ml penicillin and 50 µg/ml streptomycin. To ensure differentiation of the cells to the smooth muscle-like phenotype, cells were grown to confluency and incubated in medium containing 0% serum for 24 h prior to all experiments.

### Immunofluorescence imaging and proximity ligation assays

Cells were fixed and stained as previously described [44]. Nuclei were stained with 4,6-diamidino-2-phenylindole (DAPI, Sigma), filamentous actin was stained with Alexa Fluor® 568 and Alexa Fluor® 488 phalloidin (1∶3000, Invitrogen). Cells were examined with an Eclipse TE2000-E fluorescence microscope (Nikon, Melville, NY) equipped with a CCD camera and using filters optimized for double-label experiments. For Triton X-100 extraction, cells were incubated in PIPES/EGTA/MgCl_2_ (PEM) buffer (80 mM PIPES, pH 6.8, 1 mM EGTA, 1 mM MgCl_2_, 4% PEG) containing 0.25% Triton X-100 with gentle agitation at 37°C before fixing. For proximity ligation assays (PLA) using the Duolink in situ PLA kit (Olink, Uppsala, Sweden), coverslips were stained with the primary antibodies, washed, and further processed essentially according to the manufacturer's instructions. In brief, the coverslips were incubated with the secondary oligonucleotide-linked antibodies provided in the kit. The oligonucleotides bound to the antibodies were hybridized, ligated, amplified, and detected using a fluorescent probe. Dots were detected and counted using NIS Elements AR 2.30 software (Nikon). Images were processed with Photoshop CS3 software (Adobe Systems, Mountain View, CA).

### Cell extracts

Prior to preparation of cell lysates, cells were either stimulated with DPBA (3 mM) or with FCS (10%) for the indicated times, or left unstimulated. For cytochalasin D (CytoD) or MEK inhibitor U0126 experiments, cells were pretreated for 60 minutes, and then stimulated or left unstimulated for another 60 minutes. To prepare whole cell extracts, plates were washed with ice-cold PBS and then scraped off in lysis buffer (50 mmol/L NaCl, 3 mmol/L MgCl_2_, 1 mmol/L dithiothreitol and 0.5% Nonidet-P40 in a 10 mmol/L sodium phosphate buffer, pH 8.0) supplemented with protease inhibitor cocktail (Roche, Indianapolis, IN). Cells were lysed on ice for 30 min. Lysates were cleared by centrifugation (16,000 rcf, 10 minutes at 4°C). For Triton X-100 soluble and insoluble cell extracts, plates were washed once with either precooled (4°C) or prewarmed (37°C) PEM buffer. Cells were scraped off in prewarmed PEM buffer with 0.5% Triton X-100 and incubated at 4°C or at 37°C for 3 minutes. Cells were then pelleted by centrifugation (400 rcf at 4°C or at room temperature) for 2 minutes. The supernatant was collected and the pellet was resuspended in sample buffer ( = Triton X-100 insoluble fraction). Proteins in the supernatant ( = Triton-X soluble fraction) were precipitated over night after adding 2.5 volumes of Ethanol. Precipitated protein was pelleted by centrifugation (16,000 rcf, 10 minutes at 4°C) and resuspended in sample buffer.

### Immunoprecipitation and western blot

For immunoprecipitation experiments, A7r5 lysates were pre-cleared with empty beads and subjected to immunoprecipitation with a rabbit polyclonal anti-caveolin-1 antibody (Cell signaling) cross-linked to Protein G-dynabeads® (Invitrogen) at 4°C for 3 hours. The immobilized antigen-antibody complexes were washed three times with lysis buffer and eluted in sample buffer. Proteins in the samples were separated on 10% SDS polyacrylamide gels according to standard procedures. For western blot analysis, proteins on SDS gels were transferred onto nitrocellulose membranes (Whatman, Florham Park, NJ). Bound proteins were detected with specific primary antibodies and appropriate secondary antibodies. Bands were visualized on an Odyssey® infrared imaging system (LI-COR). Densitometry analysis was perfomed with the Odyssey 2.1 software. Phospho-ERK1/2 was normalized to total ERK1/2 co-stained on the same membranes, phospho-caldesmon was normalized to total caldesmon on duplicate membranes. Ponceau staining was used to monitor equal protein loading and transfer.

### siRNA knock down

For knock down experiments, the following siRNA oligonucleotides (Dharmacon, Lafayette, CO) were used: 5′-CGUCGAAACUGUGUGUCCCUU-3′ (antisense) and 3′-GGGACACACAGUUUCGACGUU-5′ (sense) directed against caveolin-1 and 5′-UUGUAGAUCACGUAUUUGCUU-3′ (antisense) and 5′-GCAAAUACGUGAUCUACAAUU-3′ (sense) directed against caveolin-2 ( = control). Transfection with 40 nmol/L pre-hybridized siRNA molecules was performed with Lipofectamine 2000 (Invitrogen) according to the manufacturer's instructions. Cells were processed for experiments 5 days after siRNA transfection.

### Statistical analysis

All values given in the text are mean ± SE. Differences between means were evaluated using a two-tailed Student's t-test. Significant differences were taken at the p<0.05 level.

## Supporting Information

Supporting Information S1 **Co-localization of phospho-ERK1/2 with filamentous actin and ERK1/2 distibution between caveolar and non-caveolar fractions.** (A) A7r5 cells were stimulated with either DPBA or FCS for 10 minutes, or left unstimulated, and then fixed and stained for immunofluorescence microscopy with a phospho-ERK1/2 antibody. Cells were co-stained with phalloidin to visualize actin filaments and with DAPI to visualize nuclei. Please note the filamentous ERK1/2 staining after DPBA stimulation (E and H, arrowheads), and the stronger nuclear ERK1/2 staining after FCS stimulation (I and L, arrows) compared to DPBA stimulation (E and H, arrows). Scale bar, 20 µM. (B) Unstimulated A7r5 lysates were subjected to density gradient ultracentrifugation, and fractions (numbered from top to bottom) were analyzed for total ERK1/2 in western blots. The caveolar fractions were determined by co-staining the membranes with a caveolin-1 antibody. The percentage of ERK1/2 in the caveolar fraction was determined by densitometry (n = 3).
(PDF)
