# Supplementary material for: Stimulus-Specific Activation and Actin Dependency of Distinct, Spatially Separated ERK1/2 Fractions in A7r5 Smooth Muscle Cells
Source: PLoS One. 2012 Feb 21;7(2):e30409. doi: 10.1371/journal.pone.0030409 (PMC3283592; doi:10.1371/journal.pone.0030409)
Supplement: Supporting Information S1 — Co-localization of phospho-ERK1/2 with filamentous actin and ERK1/2 distibution between caveolar and non-caveolar fractions. (A) A7r5 cells were stimulated with either DPBA or FCS for 10 minutes, or left unstimulated, and then fixed and stained for immunofluorescence microscopy with a phospho-ERK1/2 antibody. Cells were co-stained with phalloidin to visualize actin filaments and with DAPI to visualize nuclei. Please note the filamentous ERK1/2 staining after DPBA stimulation (E and H, arrowheads), and the stronger nuclear ERK1/2 staining after FCS stimulation (I and L, arrows) compared to DPBA stimulation (E and H, arrows). Scale bar, 20 µM. (B) Unstimulated A7r5 lysates were subjected to density gradient ultracentrifugation, and fractions (numbered from top to bottom) were analyzed for total ERK1/2 in western blots. The caveolar fractions were determined by co-staining the membranes with a caveolin-1 antibody. The percentage of ERK1/2 in the caveolar fraction was determined by densitometry (n = 3). (PDF) [file pone.0030409.s001.pdf]

SUPPORTING INFORMATION S1

Supplemental Material and Methods

Density gradient ultracentrifugation

For density gradient analysis of detergent-resistant material (DRM), cells were lysed in DRM lysis buffer (150 mmol/L NaCl, 25 mmol/L Tris, 10 mmol/L ATP, 5 mmol/L dithiothreitol, 5 mmol/L EDTA, 1.0% Triton X-100, pH 7.4) supplemented with protease inhibitor cocktail (Roche). After Benzonase treatment to remove DNA (50 units/ml lysis buffer for 30 min on ice), lysates were homogenized by 10 passages through a 22 gauge syringe needle, and then adjusted with Optiprep to 40%. Samples were placed on the bottom of an ultracentrifuge tube and overlayered with a discontinuous Optiprep gradient (30-5% in DRM lysis buffer). After ultracentrifugation at 200,000 x g for 4 hours in a fixed angle rotor, 11-12 fractions were collected from top and prepared for SDS-PAGE.

Figure legend

Co-localization of phospho-ERK1/2 with filamentous actin and ERK1/2 distibution between caveolar and non-caveolar fractions. (A) A7r5 cells were stimulated with either DPBA or FCS for 10 minutes, or left unstimulated, and then fixed and stained for immunofluorescence microscopy with a phospho-ERK1/2 antibody. Cells were co-stained with phalloidin to visualize actin filaments and with DAPI to visualize nuclei. Please note the filamentous ERK1/2 staining after DPBA stimulation (E and H, arrowheads), and the stronger nuclear ERK1/2 staining after FCS stimulation (I and L, arrows) compared to DPBA stimulation (E and H, arrows). Scale bar, 20 uM. (B) Unstimulated A7r5 lysates were subjected to density gradient ultracentrifugation, and fractions (numbered from top to bottom) were analyzed for total ERK1/2 in western blots. The caveolar fractions were determined by co-staining the membranes with a caveolin-1 antibody. The percentage of ERK1/2 in the caveolar fraction was determined by densitometry (n=3).

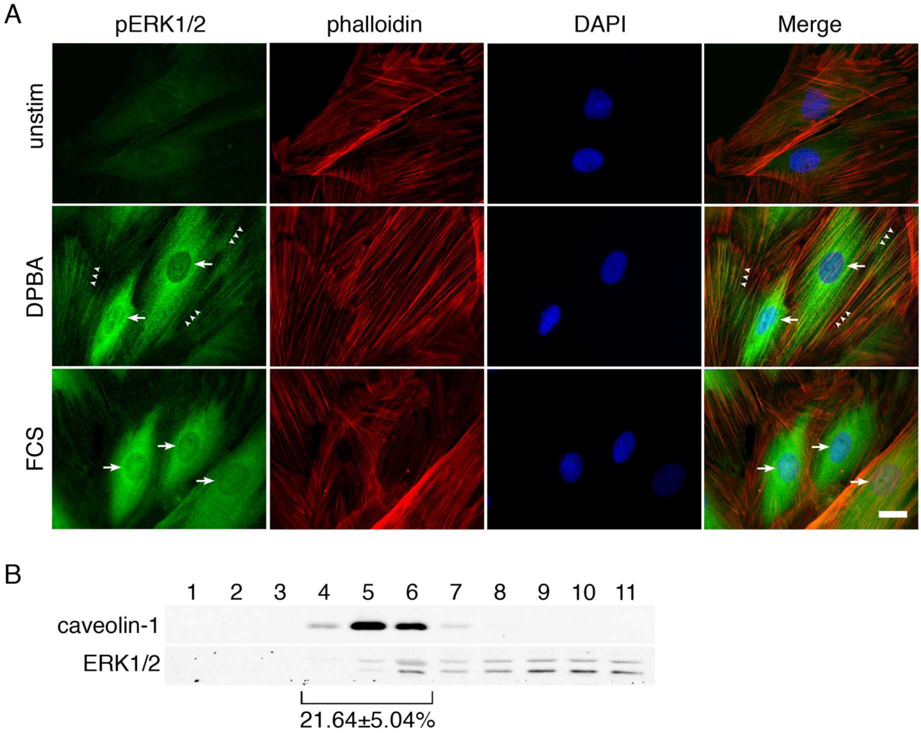
A
pERK1/2
phalloidin
DAPI
Merge
unstim
DPBA
FCS
B
1
2
3
4
5
6
7
8
9
10
11
caveolin-1
ERK1/2
21.64±5.04%
